# Supplementary material for: Modified Bose-Einstein condensation in an optical quantum gas
Source: Nat Commun. 2021 Sep 30;12:5749. doi: 10.1038/s41467-021-26087-0 (PMC8484613; doi:10.1038/s41467-021-26087-0)
Supplement: Supplementary file 1 — Supplementary Information [file 41467_2021_26087_MOESM1_ESM.pdf]

Supplementary Information

Modified Bose-Einstein condensation in an optical quantum gas

Mario Vretenar, Chris Toebes, and Jan Klaers*
Adaptive Quantum Optics (AQO), MESA+ Institute for Nanotechnology, University of Twente, PO Box 217, 7500 AE Enschede, Netherlands

In this Supplementary Information, we present a theoretical model that describes the Bose-Einstein condensation of photons in an environment with controlled dissipation and feedback and qualitatively reproduces the results observed in our experiments.

An illustration of our model is given in Supplementary Fig. 1. The wavefunction of the condensate is described by a single complex-valued amplitude ψ. This amplitude is affected by both the interaction with the optical medium, i.e. absorption and emission events, and the interaction with the environment. For the optical medium, we assume the validity of the Kennard-Stepanov law. We choose a particular implementation of the Kennard-Stepanov law, in which the emission coefficient of the medium is assumed to be frequency-independent and the absorption spectrum is assumed to increase exponentially with the frequency of the light. This corresponds approximately to the experimental conditions in our experiment. Taking this into account, we expect that the time evolution of the condensate can be described by the following dissipative Schrödinger equation:

$$\frac{i\dot{\psi}}{\psi} = \omega_0 + \omega_r + \frac{i}{2}\left(\Gamma_{\mathrm{em}} - \Gamma_{\mathrm{abs}}\,\mathrm{e}^{\frac{\hbar(\dot{\theta}-\omega_0)}{kT}}\right) - \frac{i}{2}\Gamma_{\mathrm{env}}(1 - r(\dot{\theta}))\,. \tag{5}$$

Here, ω_0 denotes the frequency of a condensate at rest, while ω_r accounts for a possible non-vanishing kinetic energy of the condensate. Γ_{em} and Γ_{abs} denote the emission and absorption rates. The particle exchange with the environment is described by the parameter Γ_{env}, which corresponds to the rate of particles that are emitted by the condensate to the Mach-Zehnder interferometer, and the reflection amplitude $r(\dot{\theta})$, which is a complex-valued function that describes magnitude and phase delay of the feedback created by the closed arm(s) of the interferometer. By describing the interferometer feedback as a complex multiple of the instantaneous wavefunction we implicitly have performed a slowly varying amplitude approximation for the time evolution of the condensate, i.e., we assume that the response time of the environment τ_{env} is so small that $|\dot{\psi}|/|\psi| \ll \tau_{\mathrm{env}}^{-1}$. Here, τ_{env} corresponds to the time the photons need to travel back and forth through the Mach-Zehnder interferometer. Another approximation concerns the absorption (Γ_{abs}) and emission rates (Γ_{em}), which we assume to be constant at the initial formation of the condensate. This essentially means that eq. (5) only correctly describes the time evolution of sufficiently small condensates. The growth rate for the condensate population and the time evolution of the phase directly follow from eq. (5) by performing a transformation $\psi = \sqrt{n}\mathrm{e}^{-i\theta}$ with real-valued functions $n = n(t)$ and $\theta = \theta(t)$:

$$\begin{aligned}\frac{\dot{n}}{n} &= \Gamma_{\mathrm{em}} - \Gamma_{\mathrm{abs}}\,e^{\frac{\hbar(\dot{\theta}-\omega_0)}{kT}} - \Gamma_{\mathrm{env}}(1 - \mathrm{Re}[r(\dot{\theta})]) \\ \dot{\theta} &= \omega_0 + \omega_r - \Gamma_{\mathrm{env}}\,\mathrm{Im}[r(\dot{\theta})]/2\,.\end{aligned} \tag{6}$$

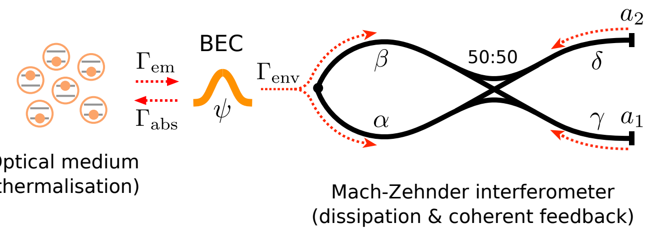

Supplementary Fig. 1. **Theoretical model.** Our model describes the Bose-Einstein condensation of photons in an environment with controlled dissipation and feedback. A thermalisation process is induced by absorption and emission processes in an optical medium. This competes with the coherent feedback from the environment, which is modelled as a Mach-Zehnder interferometer. The outputs of the interferometer are assumed to be either open or closed, which creates a varying degree of dissipation and feedback.

In our experiments, the probabilistic character of the condensation process manifests itself in the fact that the condensate state can vary from shot to shot. In our model, we can introduce this kind of randomness by converting the growth rate function $g = \dot{n}/n$ in eq. (6) into a probability distribution. To do this, we define the probability for the occurrence of a condensate with frequency $\dot{\theta}$ as $p(\dot{\theta}) = \exp(g(\dot{\theta})\,t^*)/Z$, where $g(\dot{\theta})$ is the growth rate of the state, t^* is an effective time parameter, and Z is a normalization parameter such that $\int p(\dot{\theta})d\dot{\theta} = 1$. Furthermore, an expectation value for an observable Q can be defined as $\langle Q\rangle = \langle Q\rangle_{t^*} = \int p(\dot{\theta})Q(\dot{\theta})d\dot{\theta}$. Similar to the temperature in thermal averages, the time parameter t^*

* j.klaers@utwente.nl

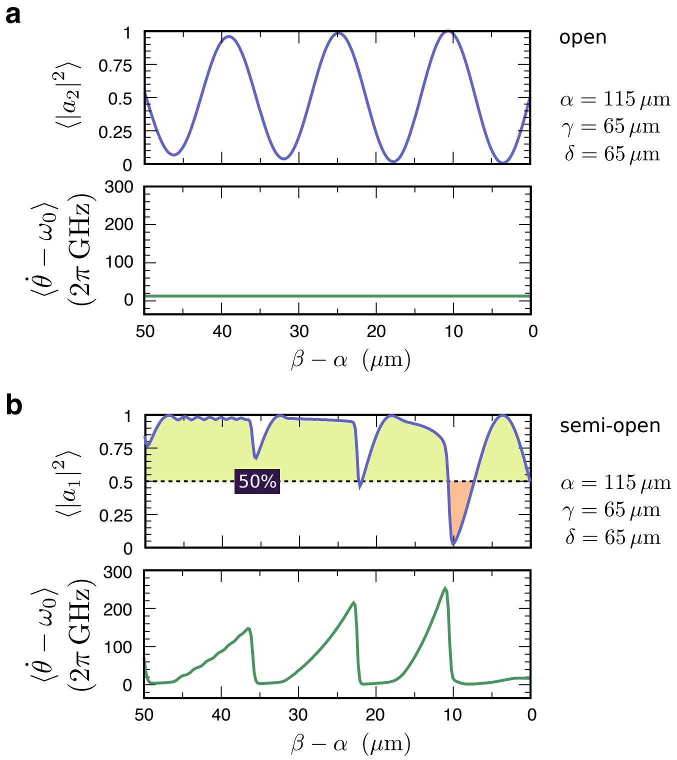

Supplementary Fig. 2. **Photon BEC in an open and semi-open Mach-Zehnder interferometer (MZI).** Theoretically expected relative photon density in the output arms and mode frequency as a function of the optical path length difference. **a** Open MZI. **b** Semi-open MZI. Parameters used to calculate these graphs are $\hbar\omega_0 = 2.1\,$eV, $m = 6.8 \cdot 10^{-36}\,$kg, $T = 300\,$K, $D_0 = 10\,\mu$m, $\Delta d = 4.5\,$nm, $\Gamma_{\rm abs} = 100\,$GHz, $\Gamma_{\rm env} = 6.3\,$GHz, and $t^* = 4.75\,$ns. All other relevant parameters are given in the figures. Note that some parameters such as $\Gamma_{\rm em}$ have no influence on the calculation of the expectation values shown here.

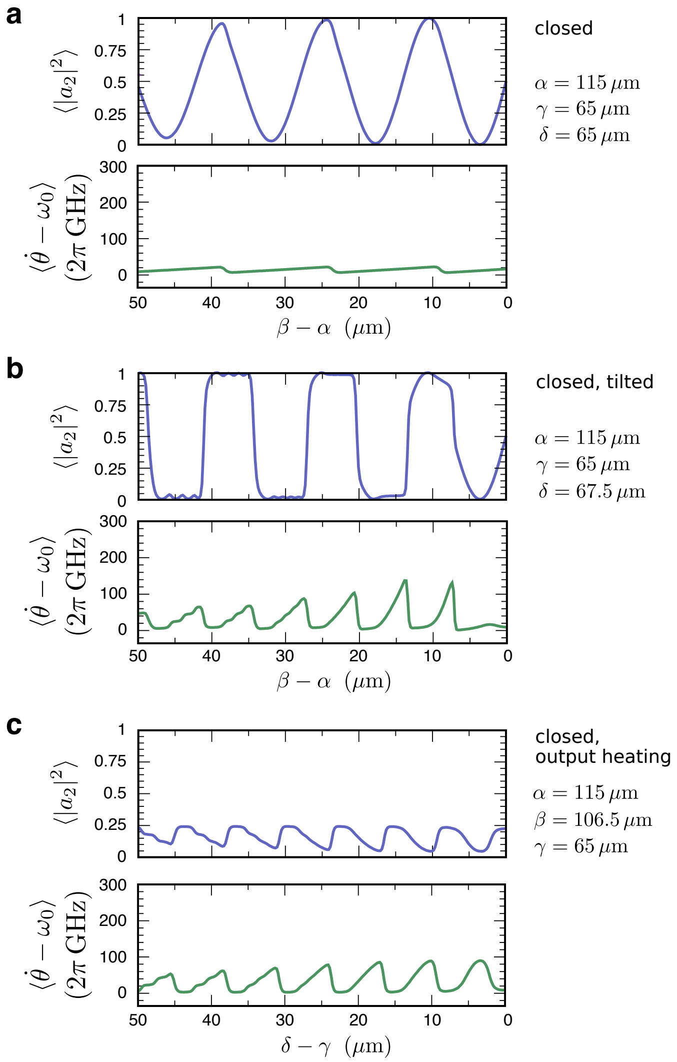

Supplementary Fig. 3. **Photon BEC in a closed Mach-Zehnder interferometer (MZI).** Theoretically expected relative photon density in the output arms and mode frequency as a function of the optical path length difference. **a** Closed MZI in plane-parallel configuration. **b** Closed MZI in tilted configuration. **c** Closed MZI, for which the optical path length tuning is performed in the upper output arm. Parameters used to calculate these graphs are the same as in Supplementary Fig. 2.

determines the statistical composition of the expectation value. For small values of t^*, many different states contribute almost equally to the average, while for large values of t^* the statistical average is dominated by the state that maximizes the growth rate. In the following, we use the time parameter as a global free parameter, which is chosen to obtain the best match between model and experiment. It turns out, however, that the found value of this parameter is close to the actual pulse duration.

In order to reproduce the experimental results with the help of the so-defined model, the frequency-dependent reflection amplitude $r = r(\dot\theta)$ has to be determined. For the open interferometer, this amplitude simply vanishes: $r_{\rm open} = 0$. In general, r is obtained by adding up the probability amplitudes of all possible paths through the interferometer, which both start and end at the condensate. As an intermediate step, we determine the probability amplitudes at the two outputs of the interferometer, which, assuming a perfect 50:50 beamsplitter, are given by $a_1 = (i\,{\rm e}^{ik(\alpha+\gamma)} + {\rm e}^{ik(\beta+\gamma)})/2$ and $a_2 = ({\rm e}^{ik(\alpha+\delta)} + i\,{\rm e}^{ik(\beta+\delta)})/2$. Here, α, β, γ, and δ denote the optical path lengths of the 4 interferometer arms, see Supplementary Fig. 1. The wavenumber $k = k(\dot\theta)$ of the photons propagating in the interferometer arms follows from $k = (2m\,[\dot\theta - \omega_0(1 - \Delta d/D_0)]/\hbar)^{1/2}$, where $\Delta d \simeq 4.5\,$nm denotes the height difference between the condensate location and the interferometer arms on the nanostructured surface of our mirror (drop in potential energy). With this, the frequency-dependent reflection amplitude in the semi-open interferometer (output 1 closed, output 2 open) becomes $r_{\rm semi} = -a_1 a_1$. For the closed interferometer, we obtain $r_{\rm closed} = -a_1 a_1 - a_2 a_2$.

Supplementary Figures 2 and 3 show results for the expected relative photon densities in the output arms of the interferometer $\langle|a_{1,2}|^2\rangle$ (upper graphs) and the mode frequency $\langle\dot{\theta}-\omega_0\rangle$ (lower graphs) as a function of the optical path length difference. For the open interferometer, our model reproduces the expected sinusoidal variation of the output intensity. No variation of the condensate frequency is predicted. For the semi-open interferometer, the imbalance in the switching function predicted by our model is even more pronounced than in the experimental data in Fig. 2c. For large path length differences, this imbalance increases, which suggests that, from a certain threshold value, the photon density is fully concentrated in the closed interferometer output regardless of the optical path length difference in the internal interferometer arms. In order to direct the particle flow into the closed interferometer arm, the frequency of the condensate can adapt to a certain extent, see lower graph in Supplementary Fig. 2b. For the closed interferometer, our theoretical model shows a transition between sinusoidal and rectangular switching functions, depending on the path length difference in the output arms, see Supplementary Fig. 3a, b. This is in good agreement with our experimental results shown in Fig. 3c. Qualitative agreement is furthermore obtained for the closed interferometer with optical path length tuning in the upper output arm, see Supplementary Fig. 3c and Fig. 4c in the article.
